# Endoscopic central airway recanalization to enable first line pembrolizumab treatment in a PD-L1 strongly positive non-small cell lung cancer: a case report

**DOI:** 10.1186/s13019-019-0862-6

**Published:** 2019-03-04

**Authors:** Alfonso Fiorelli, Fabio Perrotta, Mariano Mollica, Mario Santini, Fabiana Vitiello, Marina Gilli, Cecilia Calabrese, Andrea Bianco

**Affiliations:** 1 Thoracic Surgery Unit, University of Campania ‘Luigi Vanvitelli’, Naples, Italy; 2 Pneumology Unit, Department of Medicine and Health Sciences “V. Tiberio”, University of Molise, Campobasso, Italy; 3 Pneumology Unit, Department of Translational Medicine, University of Campania ‘Luigi Vanvitelli’- Monaldi Hospital, Via L. Bianchi, 80131 Naples, Italy; 4 Oncology unit, Monaldi Hospital, Via L. Bianchi, 80131 Naples, Italy; 5 Thoracic Surgery Unit, Second University of Naples Piazza Miraglia, 2I-80138 Naples, Italy

**Keywords:** Endoscopic airway recanalization, Pembrolizumab, Immunotherapy, NSCLC, PD-L1

## Abstract

**Background:**

Tracheobronchial malignant stenosis is a life-threatening condition which may cause recurrent infections due to lung atelectasis. Despite immunotherapy is less toxic than standard chemotherapy, recurrent lung infections may represent a challenge for this treatment.

We report a clinical case of a patient with metastatic squamous cell carcinoma suffering from pulmonary infections due to central airway obstruction who underwent endoscopic recanalization followed by immunotherapy.

**Case presentation:**

A 64 year-old man was referred to our attention for the management of metastatic squamous cell carcinoma obstructing the right main bronchus with recurrent pulmonary infections. Patient exhibited strong positive PD-L1 expression (> 50%). Advanced disease stage contraindicated surgical treatment. Although therapy with immune check point inhibitors was indicated as first-line treatment, recurrent pulmonary infections made it unfeasible. Therefore, we planned a combined approach including endoscopic recanalization of central airway in order to resolve lung atelectasis, and lung infection followed by immunotherapy treatment with pembrolizumab in order to avoid local and systemic disease progression.

**Conclusions:**

At 16-week follow-up, the patient was alive in stable disease with improvement of clinical condition and no signs of lung infection.

## Introduction

Recurrent pulmonary infections is a major challenge for Immune Checkpoint Inhibitors (ICIs) treatment in advanced lung cancer. Central airway malignant stenosis is a life-threatening condition which may cause severe respiratory distress and recurrent infections due to lung atelectasis [1].

Therapy with monoclonal antibodies directed against PD-1 or its corresponding ligand, PD-L1, has yielded impressive results in recent clinical trials, and it is a promising new treatment option for selected patients with advanced NSCLC [2]. Despite immunotherapy is less toxic than standard chemotherapy, recurrent lung infections may represent a limiting factor for this treatment.

Herein, we report a clinical case of a patient with metastatic squamous cell carcinoma suffering from pulmonary infections due to central airway obstruction who underwent endoscopic recanalization followed by immunotherapy.

## Case presentation

**Fig. 1:**
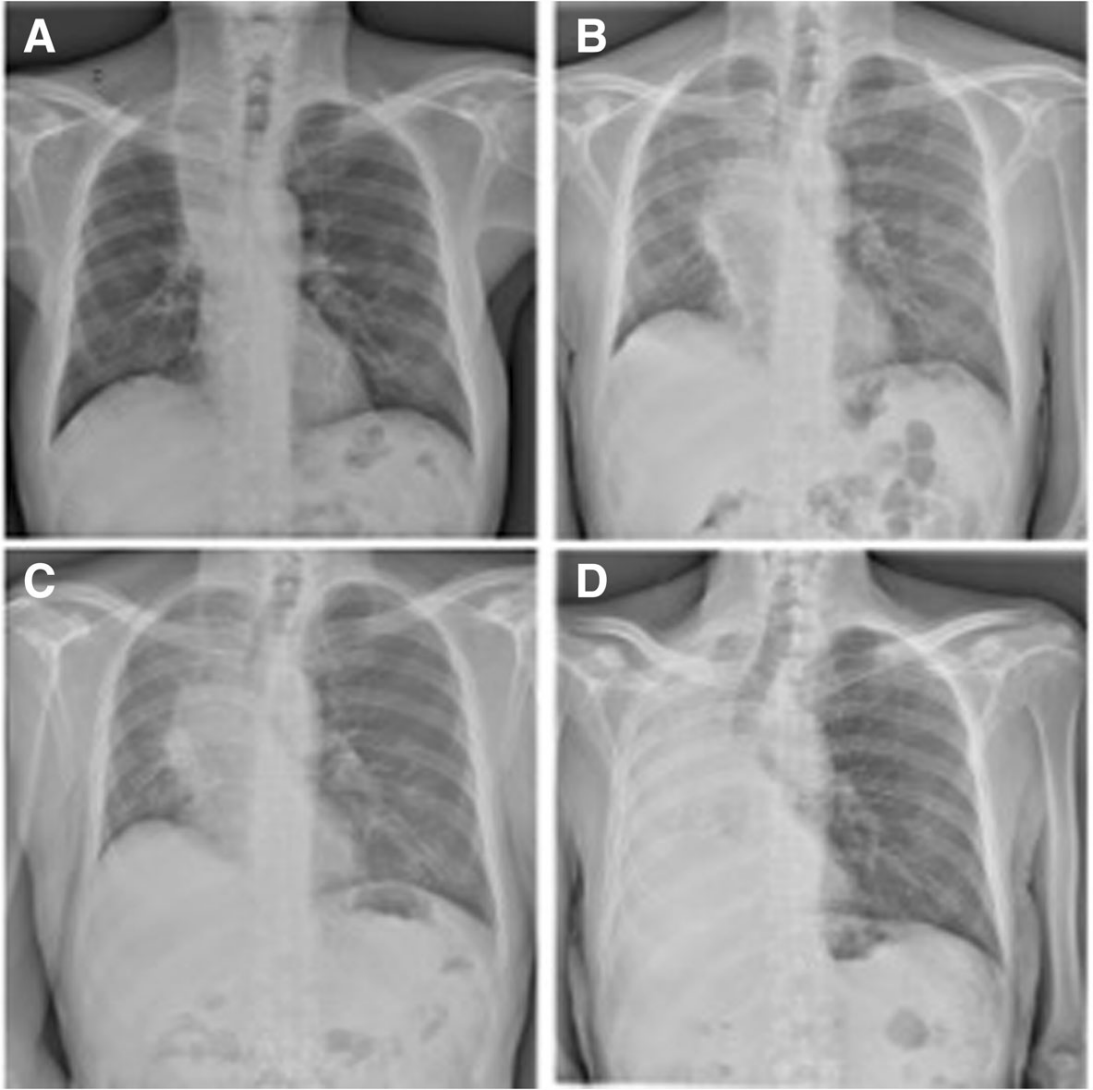
Chest x-rays showed pulmonary atelectasis involvement due to the cancer progression associated with four recurrent episodes of lung infection

**Fig. 2:**
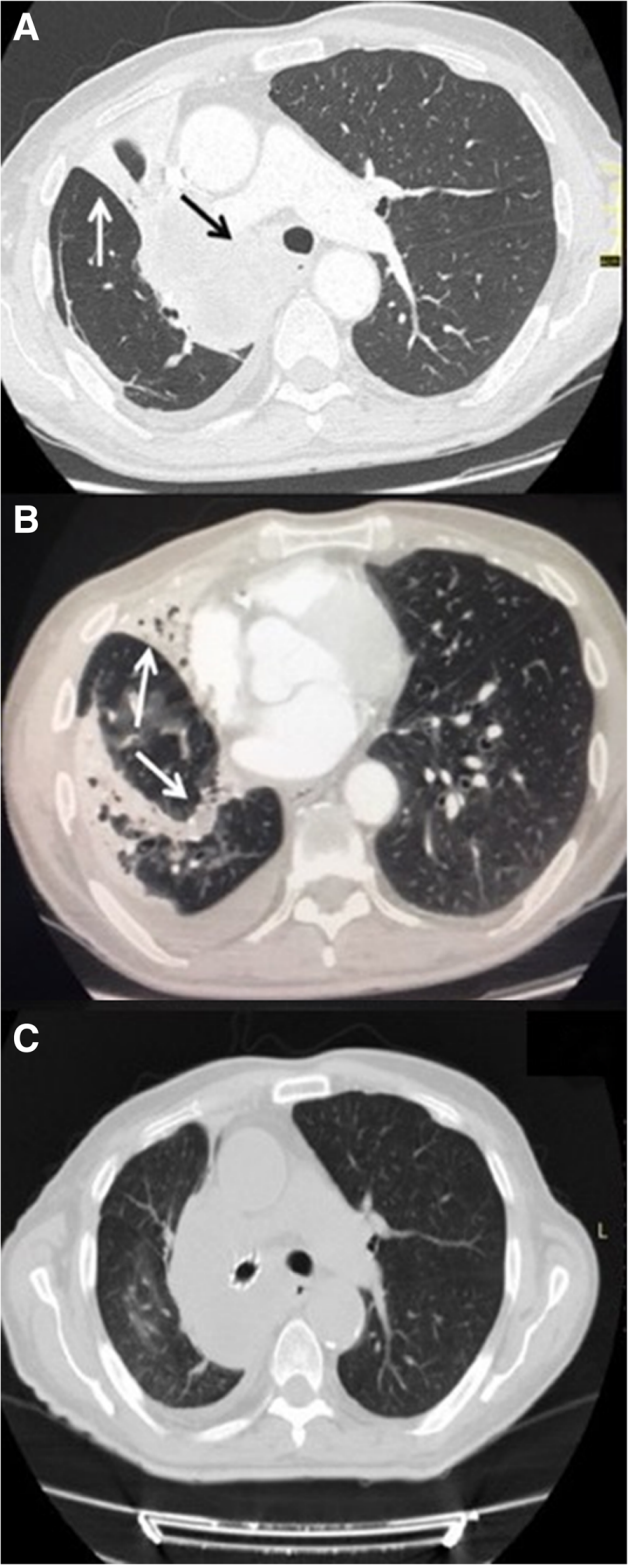
Chest computed tomography scan at the admission, showed the obstruction of the right main bronchus (black arrow) (**a**), the atelectasis of the middle lobe and the pneumonia of lower lobe (white arrow) with pleural effusion (**b**). The endoscopic recanalization with stent placement allowed to obtain the resolution of atelectasis and lung infection (**c**)

A 64 year-old male smoker was referred to our Department for the management of metastatic squamous cell carcinoma with central airway obstruction and recurrent pulmonary infections. Immunohistochemistry showed strong positive expression of PD-L1 (> 50% of tumor cells) with no EGFR or ALK genomic tumor aberrations. The patient suffered from recurrent episodes of pneumonia related to the atelectasis of right lung (Fig. 1), and exhibited severe acute respiratory distress, and poor performance status (ECOG 3). Chest CT scan performed at the admission showed a severe stricture of the right main bronchus (Fig. 2a) with atelectasis of the middle lobe and pneumonia of right lower lobe (Fig. 2b). Though Pembrolizumab was indicated as first therapeutic option, this strategy was unfeasible due to the recurrent episodes of obstructive pneumonia of right lung. Thus, after multimodal assessment the patient was scheduled for endoscopic recanalization of right main bronchus before starting ICI treatment.

**Fig. 3:**
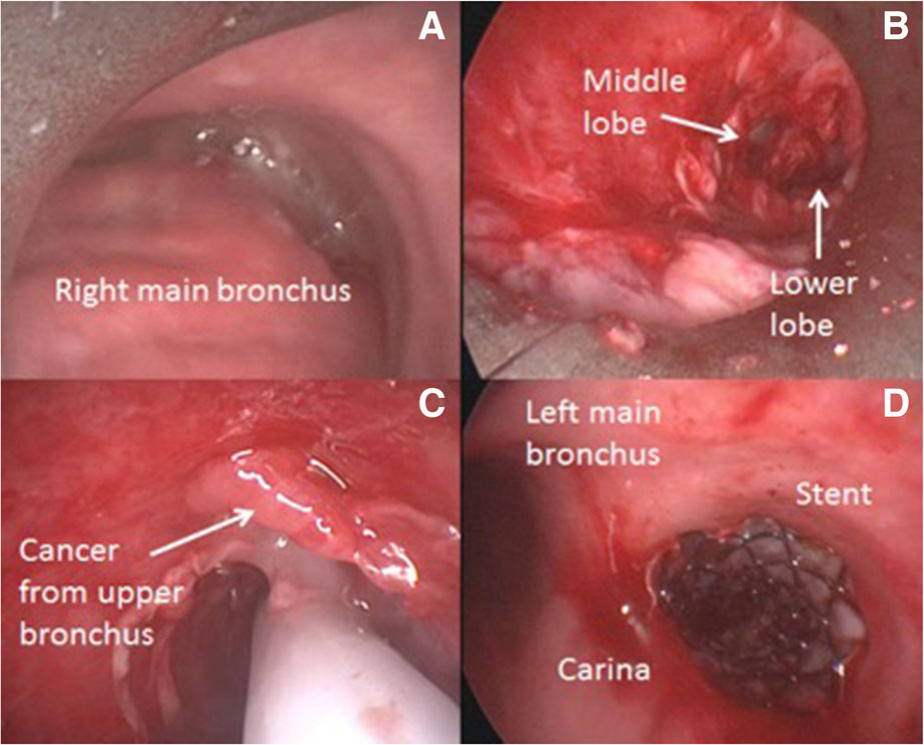
The picture edited the main steps of endoscopic recanalization. Complete obstruction of the right main bronchus from the level of the carina by an extrinsic tumor (**a**); complete recanalization of the right main bronchus, middle and lower lobe bronchus (**b**); insertion of the stent with a dedicated delivery catheter to cover the right upper lobe where the tumor originated (**c**); patency of right main bronchus after stent insertion (**d**)

**Fig. 4:**
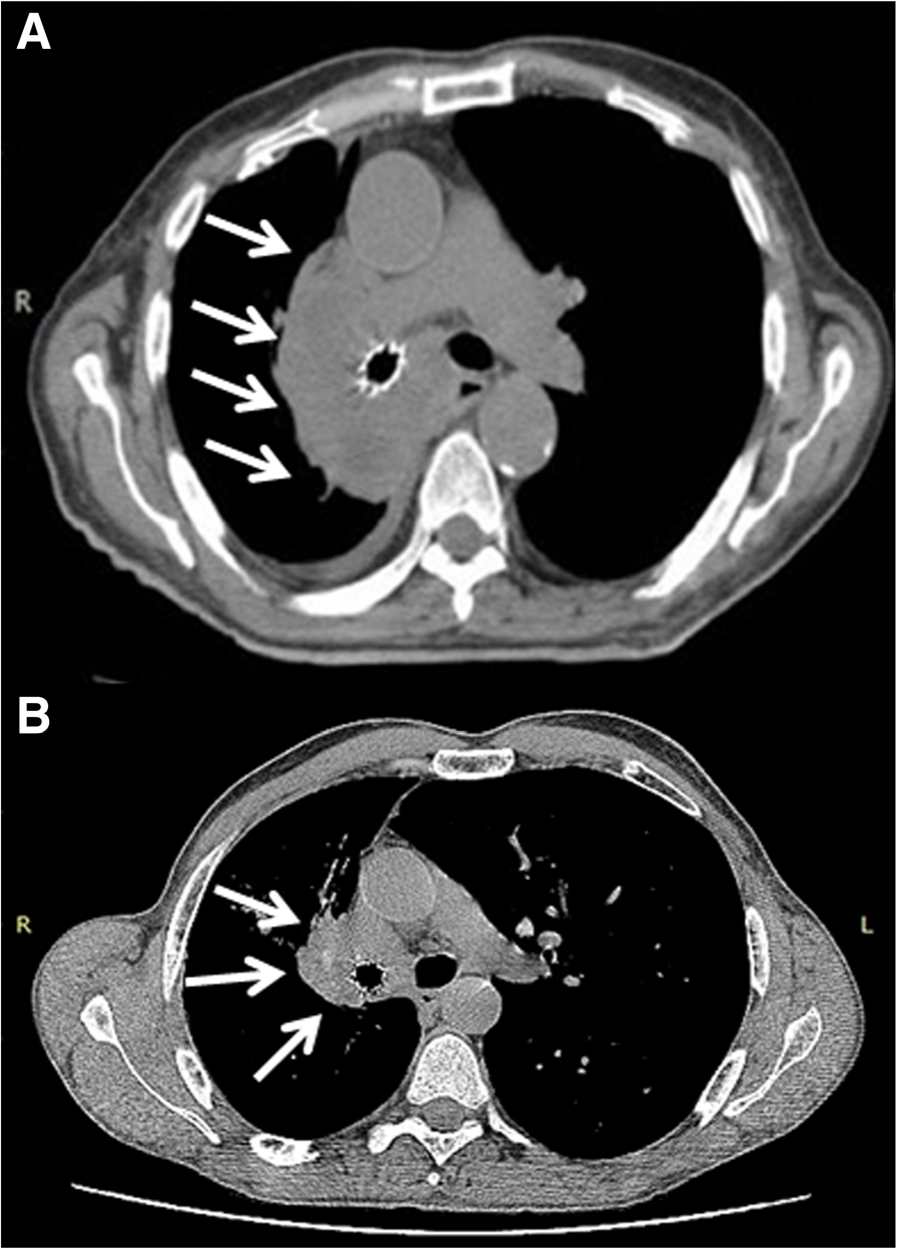
Chest CT scan performed before (Part **a**), and 16 weeks after immunotherapy (Part **b**) showed a reduction in tumor size (white arrow) without sign of lung infection

The procedure was performed under general anaesthesia; the patient was intubated with a 8,5 mm rigid bronchoscope (Stortz, Tuttlingen, Germany); the right main bronchus was completely obstructed by tumor at the level of the carina (Fig. 3a). Mechanical coring with rigid bronchoscopy, debulking with forceps, and control of bleeding with Nd:YAP laser (LokkiLis Laser-Bryan Corporation, Woburn, Mass) were used to resect the tumor and to obtain the complete recanalization of the right main bronchus and of the middle and lower bronchus (Fig. 3b). A fully covered SEMS (Tracheobronxane Silmet; Novatech SA; France, size:14 mm diameter; 40 mm length) was then inserted into the right main bronchus (Fig. 3c) to maintain airway patency (Fig. 3d). The day after the procedure the dyspnea disappeared, and patient was discharged three days later. In the following two weeks, patient did not show clinical signs of pneumonia and presented an improvement of performance status (ECOG 1); chest CT scan (Fig. 2c) confirmed the complete resolution of atelectasis and pneumonia. Therefore, he was eligible to receive Pembrolizumab 200 mg e.v. every 21 day. At 16 weeks follow-up, the patient was still alive and no further lung infections were recorded; chest CT scan (Fig. 4) showed a local reduction of tumor size without sign of lung infection.

## Conclusions

The treatment of the present case was particularly challenging due to the advanced stage of disease, and the poor clinical condition of the patient characterized by recurrent pulmonary infections, and severe respiratory distress due to central airway obstruction. Thus, we planned a new multimodal approach, not previously reported, which involved endoscopic central airway recanalization followed by ICI treatment with pembrolizumab. Our strategy was in line with Jeon et al. [3] and of Stratakos et al. [4] who showed that patients undergoing adjuvant chemotherapy and/or radiotherapy after endoscopic airway recanalization exhibited a longer survival than those who received recanalization alone.

As pembrolizumab therapy is associated with a risk of immune-mediated pneumonitis, the endoscopic recanalization of central airway was planned before starting ICI treatment in order to (i) favour the resolution of atelectasis, (ii) improve dyspnoea and general clinical status of the patient, and (iii) minimize the risk for immune-mediated pneumonitis. Following, the rationale of ICI treatment with Pembrolizumab was to prevent systemic and local disease progression; FDA and EMA have approved Pembrolizumab as first line treatment in patients with advanced NSCLC and PD-L1 strong expression, as the present, based on the results of KEYNOTE-024 study [5].

From a technical point of view, we preferred to use a SEMS rather than silicone stent due to presence of severe external compression that caused distortion and obstruction of right main bronchus [6–8]. In addition, the ICI treatment was started two weeks after the endoscopic recanalization, when a complete resolution of atelectasis and pneumonia was achieved as confirmed by improvement of patient’s clinical condition, and by chest CT scan findings.

Finally, our report proposes a new strategy in the management of advanced lung cancer patients with recurrent infections due to central airway obstruction involving local endoscopic airway recanalization followed by ICIs treatment. Obviously, our impression should be corroborated by larger experiences, before drawing definitive conclusions.

## Abbreviations

ALK: Anaplastic lymphoma kinase
CT: Computed tomography
EGFR: Epidermal growth factor receptor
EMA: European Medicines Agency
FDA: The Food and Drug Administration
ICIs: Immune Checkpoint Inhibitors
Nd:YAP: Neodymiumdoped yttrium aluminium perovskite
NSCLC: Non-small cell lung cancer
P.S: Performance status
PD-1: Programmed cell death 1
PD-L1: Programmed cell death ligand 1
SEMS: Self-Expandable Metallic Stent

## References

[1] Ost DE, Ernst A, Grosu HB, Lei X, Diaz-Mendoza J, Slade M, Gildea TR, MachuzakMS JCA, Toth J, Kovitz KL, Ray C, Greenhill S, Casal RF, Almeida FA, Wahidi MM, Eapen GA, Feller-Kopman D, Morice RC, Benzaquen S, Tremblay A (2015). SimoffM; AQuIRE bronchoscopy registry.Therapeutic bronchoscopy for malignant centralairway obstruction: success rates and impact on dyspnea and quality of life. Chest..

[2] Bianco A, Malapelle U, Rocco D, Perrotta F, Mazzarella G (2018). Targeting immune checkpoints in non small cell lung cancer. Curr Opin Pharmacol.

[3] Jeon K, Kim H, Yu CM, Koh WJ, Suh GY, Chung MP, Kwon OJ (2006). Rigid bronchoscopic intervention in patients with respiratory failure caused by malignant central airway obstruction. J Thorac Oncol.

[4] Stratakos G, Gerovasili V, Dimitropoulos C, Giozos I, Filippidis FT, Gennimata S, Zarogoulidis P, Zissimopoulos A, Pataka A, Koufos N, Zakynthinos S, Syrigos K, Koulouris N (2016). Survival and quality of life benefit after endoscopic Management of Malignant Central Airway Obstruction. J Cancer.

[5] Reck M, Rodríguez-Abreu D, Robinson AG, Hui R, Csőszi T, Fülöp A, Gottfried M, Peled N, Tafreshi A, Cuffe S, O'Brien M, Rao S, Hotta K, Leiby MA, Lubiniecki GM, Shentu Y, Rangwala R, Brahmer JR (2016). KEYNOTE-024 investigators. Pembrolizumab versus chemotherapy for PD-L1-positive non-small-cell lung Cancer. N Engl J Med.

[6] Fiorelli A, Accardo M, Galluccio G, Santini M (2013). Tracheobronchial amyloidosis treated by endobronchial laser resection and self-expanding Y stent. Arch Bronconeumol.

[7] Guarino C, Mazzarella G, De Rosa N, Cesaro C, La Cerra G, Grella E, Perrotta F, Curcio C, Guerra G, Bianco A (2016). Pre-surgical bronchoscopic treatment for typicalendobronchial carcinoids. Int J Surg.

[8] Fiorelli A, Caterino U, Raucci A, Santini M (2017). A conical self-expanding metallic stent for the management of critical complex tracheobronchial malignant stenosis. Interact Cardiovasc Thorac Surg.

